# Supplementary material for: Predation and fragmentation portrayed in the statistical structure of prey time series
Source: BMC Ecol. 2009 May 6;9:10. doi: 10.1186/1472-6785-9-10 (PMC2689204; doi:10.1186/1472-6785-9-10)
Supplement: Additional file 2 — Voles and related classes ODDox Documentation. ODDox documentation of the agent-based model (ALMaSS) applied by Hendrichsen et al. The documentation is started by activating main.html. [file 1472-6785-9-10-S2.zip › Vole_ODDox/tree.html]

TreeView


### ALMaSS ODDox

Voles and related classes ODDox Documentation

Crop Classes

Farm Management

References

Class List

AgroChemIndustryCerealFarm1

AgroChemIndustryCerealFarm2

AgroChemIndustryCerealFarm3

AlleleFreq

AnimalPosition

CompareState

CompareX

CompareY

ConventionalCattle

ConventionalPig

ConventionalPlant

ConvMarginalJord

Crop

CropRotation

Farm

FarmEvent

GeneticMaterial

GeneticMaterial1616

IntArray100

NoPesticideBaseFarm

NoPesticideNoPFarm

OrganicCattle

OrganicPig

OrganicPlant

Owl

PesticideTrialControl

PesticideTrialToxicControl

PesticideTrialTreatment

Population\_Manager

probe\_data

rectangle

RoeDeerInfo

Rotation

SetAside

Starter

struct\_Predator

struct\_Vole\_Adult

TALMaSSObject

TAnimal

TPredator

TPredator\_Population\_Manager

UserDefinedFarm1

UserDefinedFarm10

UserDefinedFarm11

UserDefinedFarm12

UserDefinedFarm13

UserDefinedFarm14

UserDefinedFarm15

UserDefinedFarm16

UserDefinedFarm2

UserDefinedFarm3

UserDefinedFarm4

UserDefinedFarm5

UserDefinedFarm6

UserDefinedFarm7

UserDefinedFarm8

UserDefinedFarm9

Vole\_Base

Vole\_Female

Vole\_Male

Vole\_Population\_Manager

Weasel

WinterWheat

Class Hierarchy

AlleleFreq

AnimalPosition

RoeDeerInfo

CompareState

CompareX

CompareY

Crop

SetAside

WinterWheat

CropRotation

Farm

AgroChemIndustryCerealFarm1

AgroChemIndustryCerealFarm2

AgroChemIndustryCerealFarm3

ConventionalCattle

ConventionalPig

ConventionalPlant

ConvMarginalJord

NoPesticideBaseFarm

NoPesticideNoPFarm

OrganicCattle

OrganicPig

OrganicPlant

PesticideTrialControl

PesticideTrialToxicControl

PesticideTrialTreatment

UserDefinedFarm1

UserDefinedFarm10

UserDefinedFarm11

UserDefinedFarm12

UserDefinedFarm13

UserDefinedFarm14

UserDefinedFarm15

UserDefinedFarm16

UserDefinedFarm2

UserDefinedFarm3

UserDefinedFarm4

UserDefinedFarm5

UserDefinedFarm6

UserDefinedFarm7

UserDefinedFarm8

UserDefinedFarm9

FarmEvent

GeneticMaterial

GeneticMaterial1616

IntArray100

Population\_Manager

TPredator\_Population\_Manager

Vole\_Population\_Manager

probe\_data

rectangle

Rotation

Starter

struct\_Predator

struct\_Vole\_Adult

TALMaSSObject

TAnimal

TPredator

Owl

Weasel

Vole\_Base

Vole\_Female

Vole\_Male

Class Members

File List

BoostRandomGenerators.h

croprotation.cpp

croprotation.h

farm.cpp

farm.h

farmfuncs.cpp

GeneticMaterial.cpp

GeneticMaterial.H

PopulationManager.cpp

PopulationManager.h

Predators.cpp

Predators.H

References\_ODDox.h

setaside.cpp

setaside.h

tole\_declaration.h

tov\_declaration.h

treatment.h

Vole\_all.cpp

vole\_all.h

vole\_ODDox.h

VolePopulationManager.cpp

VolePopulationManager.H

WinterWheat.cpp

WinterWheat.H

File Members
